# Victim help-seeking patterns and how they can inform future support services for victims of intimate partner violence

**DOI:** 10.3389/fsoc.2025.1694399

**Published:** 2025-11-28

**Authors:** Lucy Trafford, Ba Linh Le

**Affiliations:** 1 Law Faculty, University of Oxford, Oxford, United Kingdom; 2 Ludwig Maximilian University of Munich, Berlin, Germany

**Keywords:** help-seeking, domestic abuse, intimate partner violence, support services, victimisation

## Abstract

This research paper examines help-seeking behaviours of victims of intimate partner violence (IPV) in Germany by investigating the point at which victims tend to seek help in an abusive relationship and the types of services they most frequently seek support from. It is then considered how victims’ gender, age and number of children affect the type of support sought, as well as the impact that seeking support from different forms of services has upon the duration of abuse suffered. This paper utilises data collected from a nationally representative online survey conducted in Germany, which included 420 victims of domestic abuse. Initially, the frequency with which victims seek help from different forms of formal and informal support networks is evaluated (henceforth referred to as intervention). Statistical tests are then applied to examine how victims’ backgrounds, help-seeking behaviours, and the length of their abusive relationships influenced their choice of certain interventions. Ultimately, we found that the intervention relied on differed by gender, with women being more likely to seek informal support and male victims confiding more frequently in health practitioners. Victims who sought help from friends and family earlier were more likely to experience shorter abusive relationships. By contrast, victims that sought help from professional services were more likely to report longer relationships. The differences in help-seeking patterns suggest that victims have different needs and/or support networks available to them at different stages in abusive relationships. Challenges in leaving the relationship also reduced the likelihood of victims seeking support, with older victims the least likely to seek support from anyone and the number of victims ‘children increasing the time taken to confide in anyone. These findings indicate the importance of victims’ informal support networks being able to provide a supportive and understanding response when first approached for help by victims. This can be achieved through increasing social awareness of IPV and its consequences. Further qualitative research is required to identify victims’ reasons for reaching out to different services across abusive relationships, to understand the needs that victims need met at different points of the abusive relationship.

## Introduction

The prevalence and damaging impacts of intimate partner violence (IPV) on victims makes it particularly important to understand victims help-seeking behaviours, including when victims seek help in abusive relationships and the type of support that they seek. This paper focuses on the types of support services that intimate partner violence victims seek help from, at which point they tend to do so in abusive relationships and how these are impacted by individual and demographic factors. IPV is recognised as including physical, sexual, emotional, financial and digital abuse, as well as threats of harm between intimate partners. It has been described by the United Nations (2025) as any “pattern of behaviour […] that is used to gain or maintain power and control over an intimate partner.”

Prevalence rates of IPV are high, with the World Health Organization (WHO, 2024) finding that 27% of women have experienced IPV. This is very similar to the rate of women experiencing IPV within Germany, where this study was conducted, which has been identified as 25% (Schröttle and Müller, 2004). IPV can be lethal, with 38% of global femicides being committed by intimate partners, who pose the most significant risk of harm to females (WHO, 2024). Experiencing IPV also increases the likelihood of victims suffering from mental and physiological health issues, experiencing re-victimisation in future relationships and associated with an increased risk of suicidal ideation or behaviour (Cho et al., 2021). Additionally, it can have extensive impacts on children who are purposely or inadvertently harmed by abuse within the home, which increases their risk of future victimisation and perpetration (Radford and Hester, 2006). Yet, despite high prevalence rates and severe impacts, disclosure of IPV remains low (Lelaurain et al., 2017). Consequently, understanding the points at which victims choose to seek help and where they turn to for support is vital to adequately support victims of IPV and reduce the likelihood of future abuse (Bows, 2018; Fraga Dominguez et al., 2021).

### Help-seeking behaviours and implications

Help-seeking is a crucial step for victims experiencing IPV. It has been defined as the disclosure of one’s victimhood to gain assistance (Cho et al., 2021). Survivor theory describes it as a method of actively coping with abuse (Mansa, 2020). Seeking help can improve victims’ safety (Sanz-Barbero et al., 2022) and reduce the likelihood of future IPV, escalation and fatalities (Lysova and Dim, 2022; Meyer, 2010). It also acts as a vital step for victims considering separating from their abusive partner and can provide necessary practical support (Sanz-Barbero et al., 2022). Additionally, it can help victims to understand IPV, feel validated and recover from the mental, physical and emotional impacts of abuse. This can reduce the impact on victims’ mental health and well-being (Mansa, 2020; Sanz-Barbero et al., 2022). Ultimately, help-seeking can reduce IPV and provide victims with the necessary support to escape abusive partnerships (Cheng et al., 2022).

There are two primary sources from which victims may seek support independently or concurrently. These are formal and informal support networks (Cheng et al., 2022; Lysova and Dim, 2022; Sanz-Barbero et al., 2022). Informal support networks consist of victims’ friends, family and other social networks. Whilst formal support networks include public and private services, such as police, health and domestic abuse (DA) specific services (Cho et al., 2021; Liang et al., 2005). It is essential to understand the distinction between these methods of seeking support, as they differ in terms of the support and services they can provide. The different skills and services available from formal and informal support networks are likely to shape victims’ help-seeking strategies dependent upon their need (Cho et al., 2021).

Informal networks can provide victims with a more personalised understanding of their experiences, situations and resources (Meyer, 2010). In comparison, formal support services are likely to have a better understanding of the complexities of abuse, which informal networks may be unprepared to cope with (Sanz-Barbero et al., 2022). Formal services also tend to have greater resources to support victims and the authority to take legal action (Meyer, 2010). Subsequently, formal support services are often more adept at dealing with long-term needs, whilst informal support networks may quickly become exhausted (Meyer, 2010). Thus, identifying what services victims prefer at which point in their help-seeking journey can help us to better understand victims’ needs and how these may intersect with their help-seeking practices. It is essential to understand the relationship between these types of support, with various studies finding that responses received from informal support networks can shape if, when, and what formal services are approached (Peraica et al., 2021).

As well as the source approached depending on the victim’s needs (e.g., health, legal and child support), findings suggest that victims’ characteristics contribute to the likelihood of victims seeking help and the type of support networks approached (Cho et al., 2021; Lelaurain et al., 2017). These include individual, interpersonal and sociocultural factors, such as gender, age, race and socioeconomic status (Liang et al., 2005). This paper seeks to enhance our understanding of how demographic factors influence victim help-seeking, by analysing the impact of age, gender, relationship length and number of children on when victims seek support and the type of support services approached.

Fanslow and Robinson (2010) have argued that ‘to design systems and responses that are capable of actively and appropriately meeting the needs of victims’, it is vital to understand and support victims’ help-seeking journeys. Yet the reasons behind victims’ help-seeking behaviours are not fully understood, which can undermine efforts to increase support and contact with victims. Indeed, many victims do not seek critically needed help from informal or formal networks (Cho et al., 2021). This is exacerbated amongst under-researched groups, such as older and male victims (Bows, 2018; Fraga Dominguez et al., 2021). For example, few studies have considered the interaction between the victim’s gender and their support-seeking methods (Morgan et al., 2016; Robinson et al., 2021). This is despite gender intersecting with other factors, such as age, to produce individual and environmental hurdles (Bows, 2018; Fraga Dominguez et al., 2021). This perspective is essential for understanding barriers to victims’ help-seeking strategies, such as masculinity and conceptions of vulnerability. Understanding help-seeking strategies and variations in barriers concerning victims’ intersectional identities can help formal networks understand how to improve their services and raise victim awareness to improve victims’ help-seeking.

To date, studies have focused on subsets of populations (Mastrocinque et al., 2017; Robinson et al., 2021), small samples of victims (often sought from clinical settings), localised geographical areas and few or specific support services (Cho et al., 2021). This, alongside investigating a limited number of factors (Lelaurain et al., 2017) means that, often, studies are not representative or generalisable (Mastrocinque et al., 2017; Robinson et al., 2021). Furthermore, Fanslow and Robinson (2010) explain that most studies understand help-seeking as victims seeking assistance to leave the relationship, rather than focusing on how different forms of help-seeking are sought across victims’ experiences of abuse and why. To fill this gap, this research utilises a nationally representative sample with victims approached online to increase variation in participants and the types of services relied on. To truly understand help-seeking strategies, it is important to also gain perspectives from those who choose not to or are unable to seek help (Cheng et al., 2022; Robinson et al., 2021). For this reason, this study includes victims who did not seek help to understand the various, complex and intersecting barriers that victims may have faced.

### Aim

This research aims to examine the help-seeking behaviour of victims of intimate partner violence in Germany by investigating the point at which victims tend to seek help in an abusive relationship, who they seek help from and how this influences the duration of abuse. More specifically, we are interested in the following three research questions:

1. What are victims’ help-seeking patterns for when and who they reach out to?
2. Are there any gender or age-based differences in help-seeking patterns?
3. Does the number of children affect victims’ help-seeking patterns?

## Method

In this study, we analyse the results of a cross-sectional survey investigating the prevalence of intimate partner violence and the corresponding help-seeking behaviours among a nationally representative sample of 2,048 German respondents. The survey was administered over 3 days, from July 26 to July 28, 2021, 2 months after the end of the second COVID-19 lockdown in Germany (January–May 2021). As the survey was conducted during the midst of a pandemic, it is possible that the number of respondents was affected by a rise in awareness about IPV, the limited opportunities for victims to seek help from informal and formal support networks due to health and lockdown constraints, as well as services being overwhelmed (Trafford, 2022). The survey respondents were recruited from YouGov’s German online panel. To ensure national demographic representation, YouGov applied post-stratification weighting with quotas derived from the national micro-census of the German Federal Statistics Office.

The following key variables were considered aside from demographic characteristics.

### Reported victimhood

The question text was: “Prior to or during the coronavirus pandemic, did you experience controlling, coercive or threatening behaviour from a romantic partner (e.g., physical aggression, bullying, financial control, stalking) either in-person or online?.” Respondents were able to choose one out of 10 response options, indicating past year and lifetime victimhood, changes in intensity during the pandemic, relationship status and non-disclosure (“Do not know/prefer not to say”) (see Table 1). If respondents indicated experiences of IPV at some point in their lives, the following questions were shown.

**Table 1:** Unweighted differences between the sub samples: lifetime IPV victims, non-victims and non-disclosers.

Characteristic	Lifetime IPV, *N* = 420^a^	No IPV, *N* = 1,472^a^	Do not know / Prefer not to say, *N* = 156^a^	*p*-value^b^
Gender				<0.001
Male	161 (38%)	734 (50%)	74 (47%)	
Female	259 (62%)	738 (50%)	82 (53%)	
Age				<0.001
18–24	41 (10%)	100 (7%)	18 (12%)	
25–34	98 (23%)	175 (12%)	19 (12%)	
35–44	74 (18%)	206 (14%)	29 (19%)	
45–54	83 (20%)	263 (18%)	35 (22%)	
55+	124 (30%)	728 (49%)	55 (35%)	
Number of children				<0.001
None	305 (73%)	1,232 (84%)	131 (84%)	
1	54 (13%)	144 (10%)	14 (9%)	
2	48 (11%)	77 (5%)	8 (5%)	
3 or more	13 (3%)	19 (1%)	3 (2%)	
Duration of abuse: You said you experienced controlling, coercive or threatening behaviour prior to or during the pandemic, either in-person or online. How long did this behaviour last?				
0–3 months	60 (14%)	0 (NA%)	0 (NA%)	
4–6 months	47 (11%)	0 (NA%)	0 (NA%)	
7–9 months	31 (7%)	0 (NA%)	0 (NA%)	
10–12 months	47 (11%)	0 (NA%)	0 (NA%)	
1–2 years	58 (14%)	0 (NA%)	0 (NA%)	
3–4 years	38 (9%)	0 (NA%)	0 (NA%)	
5–6 years	21 (5%)	0 (NA%)	0 (NA%)	
7–8 years	19 (5%)	0 (NA%)	0 (NA%)	
9–10 years	13 (3%)	0 (NA%)	0 (NA%)	
More than 10 years	42 (10%)	0 (NA%)	0 (NA%)	
Do not know / prefer not to say	44 (10%)	0 (NA%)	0 (NA%)	
Unknown	0	1,472	156	
Duration of abuse: numerical	4.76 (2.87)	NA (NA)	NA (NA)	
Unknown	44	1,472	156	
Reach-out time: For how long did you experience controlling, coercive or threatening behaviour (from the same partner) before you spoke to someone?				
0–3 months	71 (22%)	0 (NA%)	0 (NA%)	
4–6 months	32 (10%)	0 (NA%)	0 (NA%)	
7–9 months	29 (9%)	0 (NA%)	0 (NA%)	
10–12 months	48 (15%)	0 (NA%)	0 (NA%)	
1–2 years	29 (9%)	0 (NA%)	0 (NA%)	
3–4 years	26 (8%)	0 (NA%)	0 (NA%)	
5–6 years	12 (4%)	0 (NA%)	0 (NA%)	
7–8 years	10 (3%)	0 (NA%)	0 (NA%)	
9–10 years	6 (2%)	0 (NA%)	0 (NA%)	
More than 10 years	9 (3%)	0 (NA%)	0 (NA%)	
Do not know / prefer not to say	44 (14%)	0 (NA%)	0 (NA%)	
Unknown	104	1,472	156	
Reach-out time: numerical	3.76 (2.47)	NA (NA)	NA (NA)	
Unknown	148	1,472	156	

a *n* (%); Mean (SD).

b Fisher’s Exact Test for Count Data with simulated *p*-value (based on 2000 replicates); Kruskal-Wallis rank sum test; Pearson’s Chi-squared test; Fisher’s exact test.

### Reported duration

The question text was: “You said you experienced controlling, coercive or threatening behaviour before or during the pandemic, either in-person or online. How long did this behaviour last?.” The response options were non-linear time units, starting with three-month intervals (0–3, …, 10–12 months), going over to 2-year intervals (1–2, …, 9–10 years) and ending with a plus 10-year interval (“More than 10 years”) as well as non-disclosure option (“Do not know/prefer not to say”).

### Reported uptake of support services

Victims were asked about their support system with the following question: “Which, if any, of the following support services or networks did you speak to about your experience?.” Respondents were able to choose a multiple of 12 options, including institutional services (health service, police, specialist domestic violence services, lawyer), informal networks (friends, family, place of worship), workplace networks (manager or HR rep, colleague), other and nobody.

### Reported reach-out time

The question text for this variable read: “For how long did you experience controlling, coercive or threatening behaviour (from the same partner) before you spoke to someone?.” The response options for this variable followed the same pattern as for the duration of abuse: starting with three-month intervals (0–3,…,10–12 months), going over to 2-year intervals (1–2,…,9–10 years) and ending with a plus 10-year interval (“More than 10 years”) as well as non-disclosure option (“Do not know/prefer not to say”).

To maximise the sample size, we focused on individuals who reported having experienced IPV at some point in their lives. This allowed for higher statistical power and a broader exploration of the lifetime experiences of IPV victims, although it did impact the temporal detail as the variables relating to duration and time are ordinal. The data was provided in a structured format and required no preprocessing.

### Statistical methods

The analysis consists of descriptive analysis and statistical tests of association to examine the relationships between help-seeking behaviours and victim characteristics. We included parametric and non-parametric methods to account for the small sample size. For categorical variables, absolute counts and proportions were calculated to summarise the distribution within each category. Ordinal variables were analysed using measures of central tendency such as mean and standard deviation.

To assess associations between categorical variables, chi-squared tests (expected cell count >5) and Fisher’s exact tests (expected cell count <5) were conducted. For variables with zero expected cell counts, Fisher’s exact test was performed with simulated *p*-values by Monte Carlo based on 2000 replicates (Patefield, 1981). To explore the relationships between ordinal variables, Spearman and Pearson correlation analyses were performed to account for non-linear relationships. For paired data comparisons, Wilcoxon Rank Sum Tests and *t*-tests were conducted (Calver and Fletcher, 2020). Kruskal-Wallis tests were utilised to compare the means across multiple independent groups. All statistical analyses were conducted using R version 4.3.0.

## Results

### Sample characteristics

**Figure 1:**
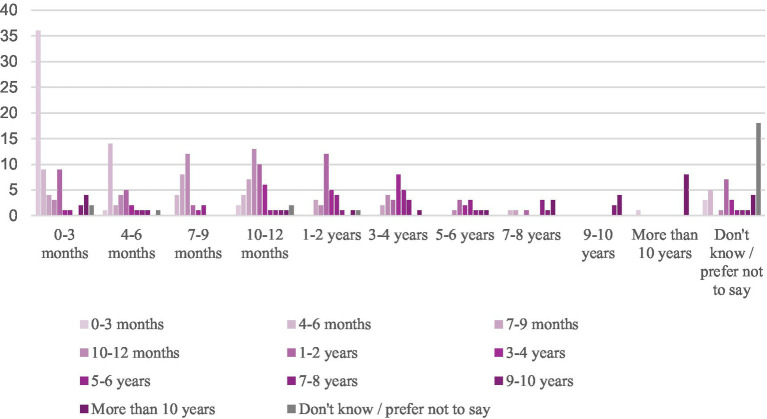
Count of respondents by reach-out time (*x*-axis) and duration of abuse (fill).

The chi-squared tests indicated multiple statistically significant demographic differences between lifetime IPV victims, non-victims, and non-disclosers (see Table 1). Lifetime IPV victims were more likely to be female (X2(2) = 17.43, *p* = <0.000), have children (X2(6) = 34.43, *p* = <0.000), and younger (X2(8) = 75.95, *p* = <0.000) with particularly higher proportions of 25–34-year-olds at risk of IPV (see Figure 1).

### Abuse duration

Half of lifetime IPV victims (57%) endured abusive experiences for up to 2 years. This finding coincided with the average of the ordinal variable, which was 4.76 (10–12 months up to 1–2 years). By contrast, more than half of victims (56%) experienced abuse by a partner for up to a year before they spoke to someone about their experiences. The average reach-out time was 3.76 (7–12 months). The Pearson’s Chi-squared test indicated that the duration of abuse was positively associated with reach-out time (X2(100) = 500.37, *p* = <0.000). Since some cell values lacked data, rendering the chi-squared test impractical, we conducted the Pearson’s correlation (*r*(264) = 0.63, *p* < 0.000) and the Spearman correlation (*r*(264) = 0.61, *p* < 0.000) as alternative measures of association on complete observations, which both pointed to statistically significant associations. This demonstrates that *longer durations of abuse are positively correlated with longer times to reach out for help.*

### Who victims seek help from

**Figure 2:**
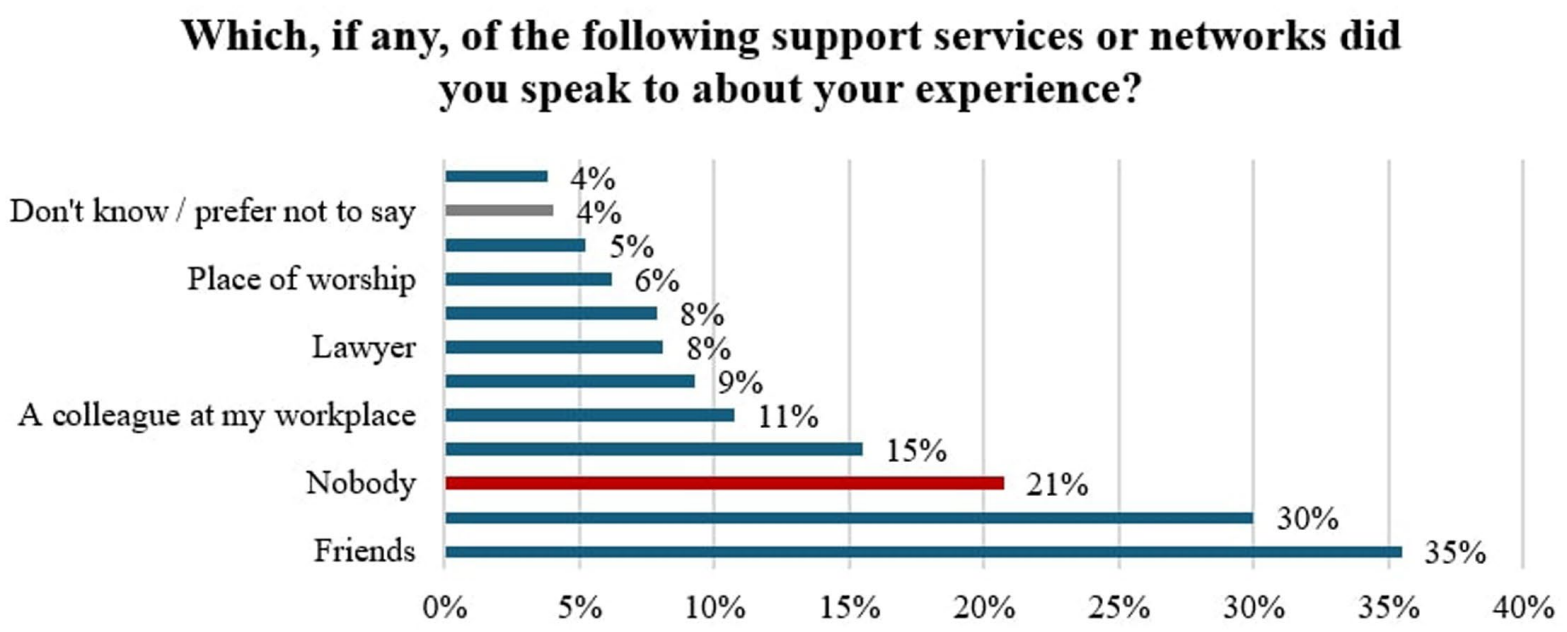
Uptake of support services sought by respondents.

**Figure 3:**
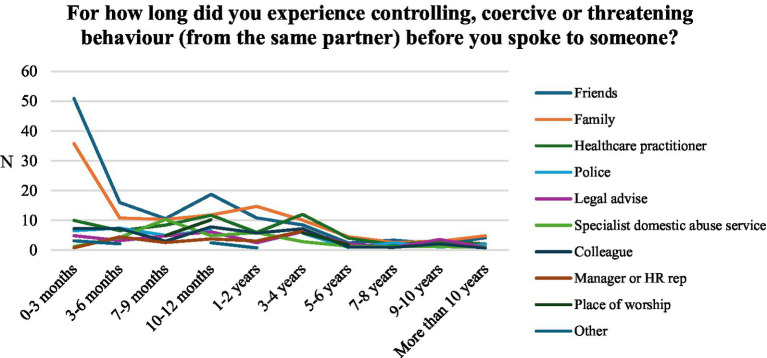
Absolute number of support service sought by respondents by time taken to reach out.

Friends and family were most often consulted by victims in this sample (see Figure 2). One in five victims who experienced IPV from a current or previous partner spoke to nobody about their experiences, with nobody representing the third highest category reported. More than one in seven victims reached out to health services, compared to less than one in ten seeking support from police and specialist DA services. Figure 3 shows the count of support services by reach-out time in the sample. Most victims reached out to friends and family within the first 4 months. Conversely, the uptake of more formal support services such as healthcare practitioners, specialist DA services, and the police appears much lower within the first 4 months and is more likely to occur later.

Who victims confide in appears to correlate with the time it took to reach out for help. To compare the reach-out times by support service, we used the Wilcoxon Test and *t*-test (see Table 2). Both tests suggest that health service, friends and the place of worship have a statistically significant association with reach-out time. The reach-out time was significantly longer for respondents who reached out to health services (*t*(95.8) = 2.5, *p* = 0.014) and a place of worship (*t*(30.2) = 2.123, *p* = 0.042). Conversely, respondents who reached out to friends reported significantly shorter reach-out times, (*t*(252.9) = −4.125, *p* < 0.000). The difference in reach-out times by legal services is only statistically significant in the Wilcoxon test (W = 4886.5, *p* = 0.047) with a higher group mean. Specialist DA services (*t*(37.86) = 1.71, *p* = 0.095) are associated with longer reach-out times at a statistical significance of 10%. Workplace networks, police, family and other support services are not significantly associated with reach-out times.

**Table 2:** Student’s *t*-test results comparing the means in reach-out time by support service (variable).

Variable	*N*	df	Group mean	Control mean	Std. Error	Lower confidence interval	Upper confidence interval	*t*	*p*-value
Health service	65	95.786	4.459	3.559	0.36	0.185	1.614	2.5	0.014
Police	39	42.23	4.029	3.722	0.491	−0.683	1.297	0.626	0.535
Specialist DA service	33	37.859	4.414	3.683	0.427	−0.134	1.595	1.712	0.095
Friends	149	252.937	3.083	4.296	0.294	−1.792	−0.634	−4.125	0
Family	126	202.975	3.66	3.825	0.316	−0.789	0.459	−0.521	0.603
Legal service	34	41.746	4.559	3.647	0.472	−0.042	1.865	1.93	0.06
Place of worship	26	30.214	4.68	3.668	0.477	0.039	1.985	2.123	0.042
Manager or HR Rep	22	25.149	4.238	3.721	0.479	−0.469	1.503	1.08	0.29
Colleague	45	55.942	4.073	3.706	0.412	−0.458	1.193	0.892	0.376
Other	16	12.558	4.923	3.703	0.995	−0.937	3.377	1.227	0.242

Due to the significant correlation between the duration of abuse and reach-out times, we suspected similar findings about the relationship between support services and duration of abuse. Wilcoxon Tests and *t*-tests confirmed the hypothesis (see Table 3). The duration of abuse was significantly longer for respondents who reached out to health services (*t*(86.475) = 2.75, *p* = 0.007; W = 11,759, *p* = 0.005) and legal services (*t*(38.35) = 2.485, *p* = 0.017; W = 7059.5, *p* = 0.018). Conversely, respondents who reached out to friends reported significantly shorter reach-out times, (*t*(295.92) = −1.935, *p* = 0.054; W = 14,413, *p* = 0.052). Aside from colleagues (*t*(53.77) = −0.414, *p* = 0.68), the tests for the other support services point to similar directions but are not significant. The results suggest that informal networks are associated with shorter durations of abuse and reach-out times, whilst more formal support services such as police and health care services are associated with longer durations of abuse and reach-out times.

**Table 3:** Student’s *t*-test results comparing the means in duration of abuse by support service (variable).

Variable	*N*	df	Group mean	Control mean	Std. Error	Lower 95% confidence interval	Upper 95% confidence interval	*t*	*p*-value
Health service	65	86.475	5.656	4.584	0.39	0.296	1.847	2.748	0.007
Police	39	45.201	4.895	4.743	0.503	−0.861	1.165	0.302	0.764
Specialist DA service	33	37.454	4.742	4.759	0.48	−0.99	0.955	−0.036	0.971
Friends	149	295.916	4.387	4.971	0.302	−1.178	0.01	−1.935	0.054
Family	126	212.433	4.67	4.797	0.325	−0.768	0.513	−0.392	0.695
Legal service	34	38.353	5.939	4.644	0.521	0.24	2.35	2.485	0.017
Place of worship	26	30.071	4.88	4.749	0.475	−0.838	1.1	0.275	0.785
Manager or HR Rep	22	25.675	4.81	4.755	0.451	−0.872	0.981	0.121	0.904
Colleague	45	53.773	4.595	4.778	0.443	−1.071	0.704	−0.414	0.68
Other	16	13.665	5.071	4.746	0.952	−1.721	2.372	0.342	0.738
Do not know	87	103.451	5.169	4.652	0.413	−0.302	1.335	1.252	0.213
Nobody	17	6.184	5.714	4.74	1.219	−1.986	3.935	0.8	0.454

**Figure 4:**
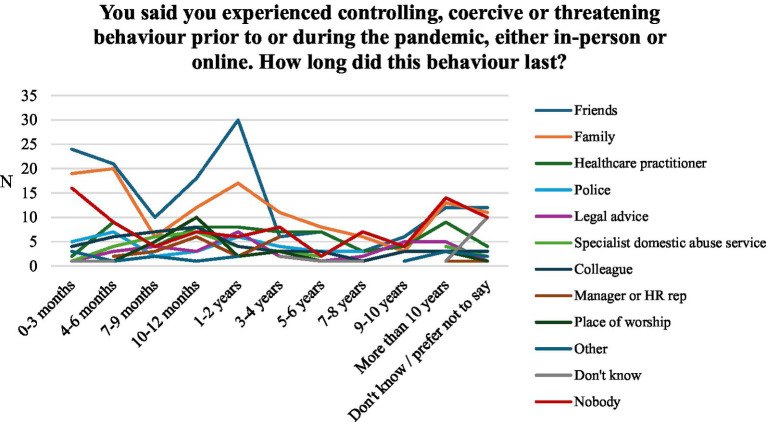
Absolute number of support service sought by respondents by duration of abuse.

Lastly, we were interested in whether certain support services were associated with a higher or lower uptake of support services. For this purpose, we created a variable counting the number of support services by respondents. Table 4 shows the results of the *t*-tests comparing the means in the count of support services by support service. All results are statistically significant, with group means of around 2 pointing to the uptake of at least 2 services. Hence, victims seeking help are likely to seek help from multiple areas. However, higher *t*-values of friends (*t*(224.75) = 10.73, *p* < 0.000) and family (*t*(195.745) = 10.29, *p* < 0.000) suggest more significant differences between the means of the groups relative to the variability within each group compared to other support services. Reaching out to health services is also associated with a high *t*-value (*t*(73.48) = 8.24, *p* < 0.000) but shows a larger group mean, suggesting a higher uptake of support services compared to friends and family (see Figure 4).

**Table 4:** Student’s *t*-test results comparing the means in count of support services by support service (variable).

Variable	*N*	df	Group mean	Control mean	Std. Error	Lower confidence interval	Upper confidence interval	*t*	*p*-value
Health service	65	73.479	2.723	1.065	0.201	1.257	2.059	8.244	0
Police	39	40.741	2.821	1.168	0.294	1.059	2.246	5.62	0
Specialist DA service	33	34.381	2.576	1.214	0.307	0.737	1.985	4.433	0
Friends	149	224.748	2.168	0.856	0.122	1.071	1.553	10.73	0
Family	126	195.745	2.238	0.929	0.127	1.058	1.561	10.287	0
Legal service	34	34.711	3.294	1.148	0.324	1.488	2.805	6.619	0
Place of worship	26	26.565	2.692	1.231	0.337	0.77	2.153	4.338	0
Manager or HR Rep	22	22.207	2.545	1.254	0.358	0.55	2.033	3.612	0.002
Colleague	45	48.003	2.867	1.136	0.259	1.21	2.252	6.681	0
Other	16	15.738	2.438	1.277	0.392	0.329	1.992	2.963	0.009

### Gender differences

Statistical tests (Pearson’s Chi-squared Test, Fisher’s Exact Test, Wilcoxon rank sum test with continuity correction) indicate numerous gender differences (see Table 5). With regards to prevalence, female respondents were more likely to have been affected by IPV at one point in their life, whilst male respondents were more likely to have been affected in the past year [*p* < (0.000)]. This mirrors similar findings by Tjaden and Thoennes (2000). The higher lifetime prevalence of IPV amongst female victims may stem from women being more likely to experience controlling and abusive behaviours from intimate partners and, consequently, in previous relationships. Male victims are more likely to be younger than female victims (X2(4) = 9.76, *p* = 0.045) and have more children (X(3) = 10.435, *p* = 0.01). There are no statistically significant differences with regard to the duration of abuse. However, both the Chi-squared Test and the Wilcoxon rank sum test indicate that males are more likely to have taken longer than females to reach out for help (X2(10) = 21.92, *p* = 0.013; W = 10,304, *p* < 0.021).

**Table 5:** Gender differences of lifetime IPV victims.

Characteristic	Male, *N* = 161^a^	Female, *N* = 259^a^	*p*-value^b^
Victimhood: Prior to or during the coronavirus pandemic, did you experience controlling, coercive or threatening behaviour from a romantic partner (e.g., physical aggression, bullying, financial control, stalking) either in-person or online?			<0.001
Yes–it began before the pandemic and stayed at the same level throughout	24 (15%)	31 (12%)	
Yes–it began before the pandemic and worsened throughout	27 (17%)	28 (11%)	
Yes–it began before the pandemic but lessened throughout	22 (14%)	11 (4%)	
Yes–it began during the pandemic and stayed the same throughout	15 (9%)	10 (4%)	
Yes–it began during the pandemic and worsened throughout	7 (4%)	12 (5%)	
Yes–it began during the pandemic but lessened throughout	8 (5%)	5 (2%)	
I did not experience such behaviour during the pandemic, but	58 (36%)	162 (63%)	
Age			0.045
18–24	15 (9%)	26 (10%)	
25–34	49 (30%)	49 (19%)	
35–44	31 (19%)	43 (17%)	
45–54	27 (17%)	56 (22%)	
55+	39 (24%)	85 (33%)	
Number of children			0.010
None	114 (71%)	191 (74%)	
1	14 (9%)	40 (15%)	
2	25 (16%)	23 (9%)	
3 or more	8 (5%)	5 (2%)	
Duration of abuse: You said you experienced controlling, coercive or threatening behaviour prior to or during the pandemic, either in-person or online. How long did this behaviour last?			0.3
0–3 months	21 (13%)	39 (15%)	
4–6 months	16 (10%)	31 (12%)	
7–9 months	16 (10%)	15 (6%)	
10–12 months	25 (16%)	22 (8%)	
1–2 years	18 (11%)	40 (15%)	
3–4 years	16 (10%)	22 (8%)	
5–6 years	9 (6%)	12 (5%)	
7–8 years	6 (4%)	13 (5%)	
9–10 years	3 (2%)	10 (4%)	
More than 10 years	13 (8%)	29 (11%)	
Do not know / prefer not to say	18 (11%)	26 (10%)	
Duration of abuse: numerical	4.58 (2.68)	4.87 (2.97)	0.4
Unknown	18	26	
Reach-out time: For how long did you experience controlling, coercive or threatening behaviour (from the same partner) before you spoke to someone?			0.013
0–3 months	16 (13%)	55 (28%)	
4–6 months	13 (11%)	19 (10%)	
7–9 months	17 (14%)	12 (6%)	
10–12 months	23 (19%)	25 (13%)	
1–2 years	10 (8%)	19 (10%)	
3–4 years	14 (12%)	12 (6%)	
5–6 years	4 (3%)	8 (4%)	
7–8 years	6 (5%)	4 (2%)	
9–10 years	2 (2%)	4 (2%)	
More than 10 years	3 (3%)	6 (3%)	
Do not know / prefer not to say	12 (10%)	32 (16%)	
Unknown	41	63	
Reach-out time: numerical	4.10 (2.30)	3.54 (2.56)	0.021
Unknown	53	95	
I spoke about my experiences to: health services			0.6
Selected	27 (17%)	38 (15%)	
Not selected	134 (83%)	221 (85%)	
I spoke about my experiences to: police			>0.9
Selected	15 (9%)	24 (9%)	
Not selected	146 (91%)	235 (91%)	
I spoke about my experiences to: specialist domestic violence service			0.2
Selected	16 (10%)	17 (7%)	
Not selected	145 (90%)	242 (93%)	
I spoke about my experiences to: friends			0.002
Selected	42 (26%)	107 (41%)	
Not selected	119 (74%)	152 (59%)	
I spoke about my experiences to: family			0.3
Selected	44 (27%)	82 (32%)	
Not selected	117 (73%)	177 (68%)	
I spoke about my experiences to: lawyer			0.5
Selected	11 (7%)	23 (9%)	
Not selected	150 (93%)	236 (91%)	
I spoke about my experiences to: place of worship			0.4
Selected	12 (7%)	14 (5%)	
Not selected	149 (93%)	245 (95%)	
I spoke about my experiences to: manager or HR rep at my workplace			0.8
Selected	9 (6%)	13 (5%)	
Not selected	152 (94%)	246 (95%)	
I spoke about my experiences to: colleague at my workplace			0.12
Selected	22 (14%)	23 (9%)	
Not selected	139 (86%)	236 (91%)	
I spoke about my experiences to: other			0.6
Selected	5 (3%)	11 (4%)	
Not selected	156 (97%)	248 (96%)	
I spoke about my experiences to: nobody			0.7
Selected	32 (20%)	55 (21%)	
Not selected	129 (80%)	204 (79%)	
I spoke about my experiences to: do not know			0.2
Selected	9 (6%)	8 (3%)	
Not selected	152 (94%)	251 (97%)	
Count of support services	1.26 (1.26)	1.36 (1.24)	0.3

a *n* (%); Mean (SD).

b Fisher’s Exact Test for Count Data with simulated *p*-value (based on 2000 replicates); Kruskal-Wallis rank sum test; Pearson’s Chi-squared test; Fisher’s exact test.

Looking at who victims report to, female victims are significantly more likely to confide in friends compared to male victims (X2(1) = 9.401, *p* = 0.002). The findings relating to other support services are not statistically significant but suggest slight gender differences: male respondents tend to reach out to health services, specialist DA services, places of worship, colleagues, and non-disclosed support services. Conversely, female respondents tend to reach out to family members, and legal services.

### Victim age

Looking at age, older victims are more likely to have experienced longer durations of abuse (X2(40) = 71.136, *p* < 0.001; Kruskal-Wallis chi-squared = 11.755, df = 4, *p* = 0.019) and less likely to seek support from anyone (X2(4) = 22.24, *p* < 0.001). The positive association with reach-out time is not statistically significant (X2(40) = 54, *p* = 0.065; Kruskal-Wallis chi-squared = 4.163, df = 4, *p* = 0.4). With regards to support service, the findings in Table 6 suggest that older victims are more likely to speak to healthcare services (X2(4) = 9.85, *p* < 0.043) and specialist DA services (X2(4) = 20.129, *p* < 0.002). Younger people, on the other hand, are more likely to confide in managers or HR representatives at their workplace (X2(4) = 10.49, *p* < 0.028). Furthermore, the difference in means of count of support services suggests that younger victims are more likely to have spoken to more support services than older victims (Kruskal-Wallis chi-squared = 9.519, df = 4, *p* = 0.049).

**Table 6:** Age differences of lifetime IPV victims with regards to help-seeking behaviour.

Characteristic	18–24, *N* = 41^a^	25–34, *N* = 98^a^	35–44, *N* = 74^a^	45–54, *N* = 83^a^	55+, *N* = 124^a^	*p*-value^b^
Duration of abuse: You said you experienced controlling, coercive or threatening behaviour prior to or during the pandemic, either in-person or online. How long did this behaviour last?						<0.001
0–3 months	5 (12%)	14 (14%)	6 (8%)	13 (16%)	22 (18%)	
4–6 months	7 (17%)	12 (12%)	7 (9%)	6 (7%)	15 (12%)	
7–9 months	5 (12%)	11 (11%)	5 (7%)	7 (8%)	3 (2%)	
10–12 months	6 (15%)	19 (19%)	10 (14%)	5 (6%)	7 (6%)	
1–2 years	4 (10%)	17 (17%)	12 (16%)	10 (12%)	15 (12%)	
3–4 years	6 (15%)	8 (8%)	5 (7%)	7 (8%)	12 (10%)	
5–6 years	1 (2%)	5 (5%)	4 (5%)	5 (6%)	6 (5%)	
7–8 years	2 (5%)	2 (2%)	2 (3%)	10 (12%)	3 (2%)	
9–10 years	1 (2%)	1 (1%)	2 (3%)	2 (2%)	7 (6%)	
More than 10 years	0 (0%)	2 (2%)	8 (11%)	10 (12%)	22 (18%)	
Do not know / prefer not to say	4 (10%)	7 (7%)	13 (18%)	8 (10%)	12 (10%)	
Duration of abuse: numerical	3.95 (2.19)	3.96 (2.13)	5.03 (2.77)	5.21 (3.05)	5.22 (3.32)	0.019
Unknown	4	7	13	8	12	
Reach-out time: For how long did you experience controlling, coercive or threatening behaviour (from the same partner) before you spoke to someone?						0.065
0–3 months	5 (14%)	14 (17%)	10 (17%)	16 (30%)	26 (30%)	
4–6 months	5 (14%)	14 (17%)	2 (3%)	3 (6%)	8 (9%)	
7–9 months	4 (11%)	14 (17%)	3 (5%)	4 (8%)	4 (5%)	
10–12 months	6 (17%)	14 (17%)	13 (22%)	6 (11%)	9 (10%)	
1–2 years	3 (9%)	10 (12%)	5 (8%)	5 (9%)	6 (7%)	
3–4 years	5 (14%)	6 (7%)	6 (10%)	4 (8%)	5 (6%)	
5–6 years	1 (3%)	3 (4%)	2 (3%)	1 (2%)	5 (6%)	
7–8 years	2 (6%)	1 (1%)	1 (2%)	4 (8%)	2 (2%)	
9–10 years	0 (0%)	0 (0%)	3 (5%)	1 (2%)	2 (2%)	
More than 10 years	0 (0%)	0 (0%)	2 (3%)	2 (4%)	5 (6%)	
Do not know / prefer not to say	4 (11%)	7 (8%)	12 (20%)	7 (13%)	14 (16%)	
Unknown	6	15	15	30	38	
Reach-out time: numerical	3.84 (2.10)	3.36 (1.79)	4.36 (2.57)	3.80 (2.79)	3.74 (2.90)	0.4
Unknown	10	22	27	37	52	
I spoke about my experiences to: health services						0.043
Selected	2 (5%)	13 (13%)	17 (23%)	9 (11%)	24 (19%)	
Not selected	39 (95%)	85 (87%)	57 (77%)	74 (89%)	100 (81%)	
I spoke about my experiences to: police						0.9
Selected	4 (10%)	8 (8%)	5 (7%)	8 (10%)	14 (11%)	
Not selected	37 (90%)	90 (92%)	69 (93%)	75 (90%)	110 (89%)	
I spoke about my experiences to: specialist domestic violence service						0.002
Selected	2 (5%)	18 (18%)	4 (5%)	5 (6%)	4 (3%)	
Not selected	39 (95%)	80 (82%)	70 (95%)	78 (94%)	120 (97%)	
I spoke about my experiences to: friends						0.2
Selected	18 (44%)	34 (35%)	32 (43%)	29 (35%)	36 (29%)	
Not selected	23 (56%)	64 (65%)	42 (57%)	54 (65%)	88 (71%)	
I spoke about my experiences to: family						0.2
Selected	13 (32%)	24 (24%)	27 (36%)	19 (23%)	43 (35%)	
Not selected	28 (68%)	74 (76%)	47 (64%)	64 (77%)	81 (65%)	
I spoke about my experiences to: lawyer						>0.9
Selected	2 (5%)	8 (8%)	7 (9%)	6 (7%)	11 (9%)	
Not selected	39 (95%)	90 (92%)	67 (91%)	77 (93%)	113 (91%)	
I spoke about my experiences to: place of worship						0.10
Selected	6 (15%)	8 (8%)	3 (4%)	5 (6%)	4 (3%)	
Not selected	35 (85%)	90 (92%)	71 (96%)	78 (94%)	120 (97%)	
I spoke about my experiences to: manager or HR rep at my workplace						0.028
Selected	4 (10%)	10 (10%)	3 (4%)	3 (4%)	2 (2%)	
Not selected	37 (90%)	88 (90%)	71 (96%)	80 (96%)	122 (98%)	
I spoke about my experiences to: colleague at my workplace						0.2
Selected	5 (12%)	14 (14%)	10 (14%)	9 (11%)	7 (6%)	
Not selected	36 (88%)	84 (86%)	64 (86%)	74 (89%)	117 (94%)	
I spoke about my experiences to: other						0.8
Selected	0 (0%)	4 (4%)	3 (4%)	4 (5%)	5 (4%)	
Not selected	41 (100%)	94 (96%)	71 (96%)	79 (95%)	119 (96%)	
I spoke about my experiences to: nobody						<0.001
Selected	4 (10%)	8 (8%)	14 (19%)	25 (30%)	36 (29%)	
Not selected	37 (90%)	90 (92%)	60 (81%)	58 (70%)	88 (71%)	
I spoke about my experiences to: do not know						0.13
Selected	2 (5%)	7 (7%)	1 (1%)	5 (6%)	2 (2%)	
Not selected	39 (95%)	91 (93%)	73 (99%)	78 (94%)	122 (98%)	
Count of support services	1.37 (1.02)	1.44 (1.20)	1.50 (1.27)	1.17 (1.40)	1.21 (1.21)	0.049

a *n* (%); Mean (SD).

b Fisher’s Exact Test for Count Data with simulated *p*-value (based on 2000 replicates); Kruskal-Wallis rank sum test; Pearson’s Chi-squared test; Fisher’s exact test.

### Children in relationship

**Figure 5:**
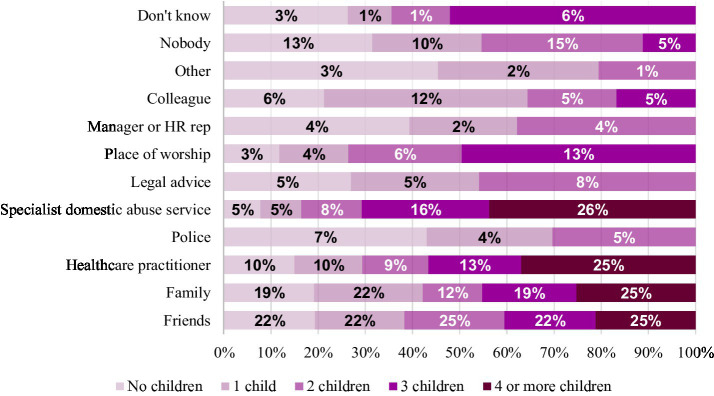
Proportions of victims reaching out to different organisations by number of children.

Lastly, we were interested in whether the number of children was associated with specific help-seeking patterns. The reach-out time was significantly correlated with the number of children (Kruskal-Wallis chi-squared = 8.282, df = 3, *p* = 0.041), suggesting longer reach-out times as the number of children increases. The results of Fisher’s exact tests indicate that respondents with more children were more likely to speak to specialist DA services [*p* = (0.021)], whilst respondents with fewer children were more likely to talk to a colleague at their workplace [*p* = (0.050)]. The remaining findings are not statistically significant but point to specific differences by number of children: respondents with no or fewer children tend to reach out to the police, lawyers, a manager or HR representative at their workplace and nobody. On the other hand, respondents with more children tend to speak to health services and someone at a place of worship (see Figure 5).

## Discussion

Overall, this study found that 79% of victims did seek support of some kind, with the type sought varying depending on the duration of abuse and the victim’s characteristics. Informal support networks such as friends (*M* = 3.08, SD = 2.43) and family members (*M* = 3.66, SD = 2.67) appear to act as vital support systems for victims in the early stages of abuse. In contrast, formal support systems such as healthcare practitioners (*M* = 4.46, SD = 2.49), specialist DA services (*M* = 4.41, SD = 2.13) and police (*M* = 4.03, SD = 2.75) are more likely to be contacted and relied upon by victims who have experienced abuse over a longer period (Triantafyllou et al., 2019). Reliance upon these formal services appears to be associated with victims reaching out to a higher number of support services, as well as with longer durations of abuse. The data indicates that victims first spoke with friends and family members about their experiences of abuse between 7–9 months and 10–12 months. Whilst formal support services were consulted between 10–12 months and 1–2 years. Earlier reach-out points were associated with shorter durations of abusive relationships, suggesting that early interventions can significantly reduce the duration of abuse experienced by victims.

### Greater reliance on informal support networks

Victims may be more likely to seek help from informal support networks, such as friends and family (Fanslow and Robinson, 2010; Meyer, 2010; Sanz-Barbero et al., 2022), as the close nature of these relationships provides victims with more personalised and nurturing support (Cho et al., 2021). Additionally, victims may fear the implications of engaging with formal services, such as their abuser being criminalised, as victims are likely to be financially, practically and emotionally affected by sanctions imposed. Indeed, less than a tenth of victims in this study contacted the police for support. This mirrors similar findings that less than 20% of victims report abuse to the police (Cho et al., 2021; Tjaden and Thoennes, 2000). Victims may also fear implications from state services, including that they or their partners will be deported, or they will be separated from their children (Hoyle, 1998). Perpetrators can play on these fears as a tactic to reduce victims’ help-seeking behaviours.

In support of this notion, this study found that victims with more children were significantly less likely to seek support from police and lawyers and more willing to contact specialist DA services. This may be because, unlike other formal networks, specialist DA services provide victims with anonymity and cannot enforce formal actions. Consequently, they allow victims to evaluate their options, enabling them to choose whether to involve other services. This suggests that campaigns may need to increase victims’ trust in formal services.

This research also found that victims who seek any help are likely to approach several networks. This may stem from a snowball effect, with informal and formal support networks recommending alternative services to victims as different providers specialise in other areas of support (Liang et al., 2005). As such, responses from informal support networks can influence victims’ future help-seeking decisions from formal services. Indeed, Peraica et al. (2021) refer to informal support as a method of ‘empowering abused women to seek professional help’. This makes it particularly important to increase and improve social awareness and understanding of IPV (Bates, 2020; Meyer, 2010).

### The diminishing roles of informal support networks

Victims may be more likely to speak with friends and family earlier in abusive relationships as these support networks often provide opportunities to discuss and evaluate romantic experiences. Women are more likely to discuss intimate relationships with their family and friends to gain advice or affirmation (Liang et al., 2005), which may contribute to females being more likely to disclose abuse to friends and family. These discussions are more likely to occur during the beginning of a relationship, which may also explain the greater rates of disclosure to friends and family earlier on in abusive relationships. In comparison, closeness and communication with these informal support networks are likely to diminish throughout abusive relationships due to controlling and abusive behaviours, as well as the practical, emotional and psychological implications of abuse (Lelaurain et al., 2017; Fraga Dominguez et al., 2021; Overstreet and Quinn, 2016). Subsequently, the impact of abuse on victims can impede help-seeking strategies in the long term.

Longer durations of abuse are positively associated with increased time taken to seek support. This may be due to the compounded impact of abuse reducing victims’ access to social support networks and making it more difficult to share experiences (Cho et al., 2021). As such, victims who experience abuse over an extended period may be more likely to seek support from formal practitioners due to reduced social networks, making formal networks a core support provider for victims who have experienced longer periods of abuse (Lelaurain et al., 2017; Morgan et al., 2016; Robinson et al., 2021).

Similarly, older victims who experienced significantly longer durations of abuse were more likely to seek support from formal services such as healthcare and specialist DA services (Cheng et al., 2022; Sanz-Barbero et al., 2022). These victims may experience more significant periods of isolation and subsequently have reduced social support networks (Bows, 2018; Fraga Dominguez et al., 2021). Support networks external to one’s family are also likely to decrease with age. Additionally, older victims may be unwilling to disclose abuse to loved ones after years of suffering (Monckton-Smith, 2021). They may seek help from healthcare providers and specialist DA services, which provide opportunities to confidentially seek support and validate their feelings. Due to changes in social perceptions and understandings of DA, younger victims may be more likely to confide in friends, family, and colleagues.

### The impacts of abuse on perceptions of self and stigmatisation

Victims who have experienced longer periods of abuse may be more likely to seek help from formal services, as abuse can have a myriad of consequences for victims’ mental and physical health (Bates, 2020). For example, the accumulation of abuse can undermine victims’ sense of self, which can make it difficult for victims to reach out (Lelaurain et al., 2017). Victims may feel that the abuse suffered reflects their failures and that they are to blame for provoking or infuriating their partner (Fraga Dominguez, bows). Consequently, victims may fear being judged, ostracised or discredited, which can reduce their willingness to disclose abuse (Lelaurain et al., 2017; Overstreet and Quinn, 2016; Robinson et al., 2021; Taylor et al., 2022). Indeed, victims internalised perspectives surrounding IPV, including embarrassment, shame and stigma, can act as barriers to help-seeking (Fraga Dominguez et al., 2021; Overstreet and Quinn, 2016). These feelings of shame and fear of stigmatisation may be compounded for older victims by generational attitudes towards IPV (Sanz-Barbero et al., 2022) which conceptualise abuse as a private matter (Bows, 2018; Fraga Dominguez et al., 2021). These internalised perspectives can reduce victims’ willingness to disclose abuse (Cho et al., 2021; Lelaurain et al., 2017).

It appears that gender can compound the stigmatisation experienced by victims, consequently reducing the likelihood of male victims seeking help (Cho et al., 2021; Lysova and Dim, 2022; Taylor et al., 2022). In line with recent studies (Peraica et al., 2021), this study found that male victims took longer to reach out for help than females. Male victims also had significantly lower rates of disclosing abuse to friends, which is likely to be related to victims’ fears of emasculation and embarrassment (Lysova and Dim, 2022). Vulnerabilities stemming from victimisation and the need to seek help can challenge masculine ideals of independence and expectations that males can protect themselves (Cook, 2009; Hamel, 2009; Taylor et al., 2022). Social constructions of masculinity can also cause male victims to fear secondary victimisation by being incorrectly labelled as the perpetrator. Again, these are fears that perpetrators have been found to exploit, which reinforce barriers to disclosure (Taylor et al., 2022). Qualitative studies have similarly found that male victims often fear being perceived as ‘weak’, ‘unmasculine’, or ‘unmanly’ due to social expectations and masculine norms (Brookes and Chałupnik, 2023), particularly in intimate relationships (Robinson et al., 2021).

Interestingly, feelings of shame and stigma may explain our finding that both older victims and male victims are more likely to seek help from organisations where their identities can remain anonymous and their experiences confidential, such as GPs and DA services. These fears may be mitigated by seeing professionals who can provide what is viewed as objective confirmation of the victim’s abusive experiences. This supports Williams and Mickelson’s (2008) findings that there is an association between victims’ feelings of shame/embarrassment and their likelihood of seeking support from providers who prioritise their anonymity. Hence, gendered and generational social perceptions and expectations regarding gender roles and relationships can act as a barrier to help-seeking for both male and female victims (Robinson et al., 2021). Victims’ perspectives of their own experience and victimisation are also likely to impact their willingness to seek help (Morgan et al., 2016). Indeed, one of the critical factors impacting victims’ likelihood of seeking help is recognition of their victimhood, which can be difficult for victims due to the social and stigmatising connotations of victimisation (Lelaurain et al., 2017; Mansa, 2020; Overstreet and Quinn, 2016; Robinson et al., 2021).

### The complexity of relationships

The association between relationship length and time taken to seek help may be influenced by increased complexity in intimate relationships as they progress. Increases in family obligations and financial interdependence can make it harder for victims to separate from partners. Financial difficulties pose problems for all victims seeking to leave their partners, as it can be challenging to develop financial independence to cover living costs (Robinson et al., 2021; Sanz-Barbero et al., 2022). As seeking support is often the first step to separating from abusive partners, as is evident from the correlation between the length of an abusive relationship and the time taken to seek help, these barriers can make it difficult for victims to reach out (Fanslow and Robinson, 2010; Lelaurain et al., 2017; Robinson et al., 2021). Seeking help may not be seen to have any tangible benefits where separating from an abuser does not appear to be a viable option (Triantafyllou et al., 2019).

These issues appear to be compounded by the number of children in the relationship and the victims’ age, as these can exacerbate barriers and introduce additional complexities to leaving abusive relationships. Financial interdependence is likely to be greater for older victims, particularly where their partner has been the primary earner, as was traditionally the case (Bows, 2018; Fraga Dominguez et al., 2021; Sanz-Barbero et al., 2022). Whilst victims with children face additional costs to meet their children’s needs, which increases for each child (Lelaurain et al., 2017).

Furthermore, IPV is often linked to traditional gender norms and sociocultural expectations which can remove or reduce women’s participation in the work environment and ability to develop their financial independence (Stark, 2007). This is an impact that is greater for women with children as childcare is traditionally associated with women’s domestic roles, and women are less likely to work after having children due to the prohibitive costs of childcare. Kleven (2022) refer to this as the child penalty. Victims with children also face the emotional cost of either leaving their children or separating children from their parent or parental role model (Lelaurain et al., 2017). Additionally, the prospect of leaving their partner may be more challenging for women with children due to societal expectations around motherhood and family stability. This reinforces Kandiyoti’s (1988) concept of the patriarchal bargain, as women may maintain their silence within gendered power structures, or not pursue support available to them, due to patriarchal expectations, such as maintaining family cohesion or social respectability.

Older victims also face care-related issues concerning adult children and their partners. Victims may be unwilling to disclose abuse to their children or formal services as their children, friends, and relatives may become aware of their situation (Fraga Dominguez et al., 2021). Older victims and perpetrators are also likely to suffer from personal frailty and illnesses, which may make them reliant on or responsible for abusive partners (Bows, 2018; Fraga Dominguez et al., 2021; Triantafyllou et al., 2019). Hence, emotional and financial impacts can increase the time taken for older victims and victims with children to seek help.

Similarly, hopelessness, such as from a lack of workable options, can make victims reluctant to seek help (Lelaurain et al., 2017). Concerningly, Beaulaurier et al. (2008) found hopelessness to be a critical factor in reducing help-seeking among older victims due to the practical difficulties of separating from their partners, living independently and the uncertainty of how much longer they would be alive (Triantafyllou et al., 2019).

### Implications

Overall, these findings suggest that it is essential to encourage victims to reach out for help as well as to bolster the informal support networks that victims can reach out to when they initially experience abuse. This is because victims appear more likely to reach out to informal support networks earlier in their abusive relationships, making it a cornerstone of support, and because seeking support at an earlier stage was significantly associated with relationship length. This suggests that informal support networks can help victims to recognise their partner’s abusive behaviour and its impact on them, as well as their options and need to remove themselves from the relationship (Sanz-Barbero et al., 2022). Consequently, it is important to increase social awareness of IPV and its consequences, a step that various countries took during lockdown to increase victims’ disclosures (Trafford, 2022). Goodmark (2018) suggests that in supporting victims, it is essential to ensure that they have social support networks, such as family and friends, who can collaborate to provide access to community-based resources, in comparison to CJS. Without these support networks, victims are likely to find it harder to seek help, which can further isolate and embed them within these abusive relationships for a more extended period.

Reliance on formal services by victims who have experienced more extended periods of abuse demonstrates that these services should be equipped to deal with victims’ various and complex needs. As ‘stigma is a product of social interaction’ (Taylor et al., 2022), it is hoped that improving social understanding of IPV could help reduce the shame and stigma that can prevent victims, particularly males and older victims, from disclosing abuse. Future research should investigate victims’ motivations for seeking help. This could help illuminate why victims seek help from different providers, as well as why the support network type (formal or informal) is likely to differ depending on the duration of abuse suffered.

### Limitations

Participation in this survey was limited to adult respondents from YouGov’s panel who spoke German. Therefore, vulnerable groups who are particularly susceptible to IPV might be underrepresented. However, a key strength of this study is that using a nationally representative sample provides information on help-seeking practices from a variety of victims who have contacted various forms of support. It also avoids issues of generalisability that are often faced by studies which are constrained to specific sub-samples of the population, or victims that have sought help from a particular type of service (Fanslow and Robinson, 2010). Second, the experience of abuse was polled by a single question. Whilst the question was worded expansively, multi-item lists have been beneficial for disclosing IPV victimhood. Finally, results relating to the duration of abuse and reach-out time should be interpreted with their ordinal nature in mind. Ordinal response options were deliberately chosen to facilitate rapid and effortless participant responses.

## Conclusion

This study found the duration of abuse and reach-out time in IPV cases to be significantly associated with help-seeking patterns and demographic factors such as gender, age and number of children. More specifically, victims were more likely to reach out to informal networks at earlier stages of the relationship and early reach-out was associated with shorter durations of abusive relationships. Where victims are unable to seek help for abuse suffered during the earlier stages of their relationship, they are likely to stay within the abusive relationship for a longer time. This may be due to the insidious nature of abuse, making it hard for victims to reach out for help due to isolation, fears of stigmatisation and being reliant on their abuser to provide for themselves and their family. This is likely to be affected by decreases in victims’ social support networks and an increase in the need for multifaceted support stemming from the physical and psychological impacts of abuse. This indicates how gender and age can intersect to create additional barriers for, particularly older, victims (Bows, 2018); a previously under-researched consideration (Sanz-Barbero et al., 2022). We recommend further research looking at the duration of abuse as an under-researched indicator of impact.

## Data Availability

The original contributions presented in the study are included in the article/supplementary material, further inquiries can be directed to the corresponding author.

## References

[ref1] Bates E. A. (2020). “No one would ever believe me”: an exploration of the impact of intimate partner violence victimization on men. Psychol. Men Masc. 21, 497–507. doi: 10.1037/men0000206

[ref2] Beaulaurier R. L. Seff L. R. Newman F. L. (2008). Barriers to help-seeking for older women who experience intimate partner violence: a descriptive model. J. Women Aging 20, 231–248. doi: 10.1080/08952840801984543, PMID: 18983109

[ref3] Bows H. (2018). “Violence against older women” in The Routledge handbook of gender and violence. (London: Routledge), 183–195.

[ref4] Brookes G. Chałupnik M. (2023). Masculinities and discourses of men’s health (Germany: Springer), 1–23.

[ref6] Calver M. Fletcher D. (2020). When ANOVA isn’t ideal: analyzing ordinal data from practical work in biology. Am. Biol. Teach. 82, 289–294. doi: 10.1525/abt.2020.82.5.289

[ref7] Cheng S.-Y. Wachter K. Kappas A. Brown M. L. Messing J. T. Bagwell-Gray M. . (2022). Patterns of help-seeking strategies in response to intimate partner violence: a latent class analysis. J. Interpers. Violence 37, NP6604–NP6632. doi: 10.1177/0886260520966671, PMID: 33084471

[ref8] Cho H. Kwon I. Shamrova D. Seon J. (2021). Factors for formal help-seeking among female survivors of intimate partner violence. J. Fam. Violence 36, 143–152. doi: 10.1007/s10896-019-00107-6

[ref9] Cook P. W. (2009). Abused men: the hidden side of domestic violence. Ukraine: ABC-CLIO.

[ref11] Fanslow J. L. Robinson E. M. (2010). Help-seeking behaviors and reasons for help seeking reported by a representative sample of women victims of intimate partner violence in New Zealand. J. Interpers. Violence 25, 929–951. doi: 10.1177/0886260509336963, PMID: 19597160

[ref12] Fraga Dominguez S. Storey J. E. Glorney E. (2021). Help-seeking behavior in victims of elder abuse: a systematic review. Trauma Violence Abuse 22, 466–480. doi: 10.1177/1524838019860616, PMID: 31291837

[ref9004] Goodmark L. (2018). Decriminalizing Domestic Violence: A Balanced Policy Approach to Intimate Partner Violence. Gender and Justice. Oakland, California: University of California Press.

[ref14] Hamel J. (2009). Toward a gender-inclusive conception of intimate partner violence research and theory: part II–new directions. Int. J. Mens Health 8, 41–59. doi: 10.3149/jmh.0801.41

[ref15] Hoyle C. (1998). Negotiating domestic violence: police, criminal justice and victims. Oxford: Oxford University Press.

[ref16] Kandiyoti D. (1988). Bargaining with patriarchy. Gender Soc. 2, 274–290. doi: 10.1177/089124388002003004

[ref9003] Kleven H. (2022). The Geography of Child Penalties and Gender Norms: A Pseudo-Event Study Approach. w30176. Cambridge, MA: National Bureau of Economic Research. doi:10.3386/w30176

[ref17] Lelaurain S. Graziani P. Monaco G. L. (2017). Intimate partner violence and help-seeking. Eur. Psychol. 22, 263–281. doi: 10.1027/1016-9040/a000304

[ref18] Liang B. Goodman L. Tummala-Narra P. Weintraub S. (2005). A theoretical framework for understanding help-seeking processes among survivors of intimate partner violence. Am. J. Community Psychol. 36, 71–84. doi: 10.1007/s10464-005-6233-6, PMID: 16134045

[ref19] Lysova A. Dim E. E. (2022). Severity of victimization and formal help seeking among men who experienced intimate partner violence in their ongoing relationships. J. Interpers. Violence 37, 1404–1429. doi: 10.1177/0886260520922352, PMID: 32469671

[ref20] Mansa M. (2020). Coping strategies of women intimate partner violence survivors: perspectives of service providers. [PhD thesis]. Saskatoon, Canada: University of Saskatchewan.

[ref21] Mastrocinque J. M. Thew D. Cerulli C. Raimondi C. Pollard R. Q. Jr. Chin N. P. (2017). Deaf victims’ experiences with intimate partner violence: the need for integration and innovation. J. Interpers. Violence 32, 3753–3777. doi: 10.1177/0886260515602896, PMID: 26371087

[ref22] Meyer S. (2010). “Responding to intimate partner violence victimisation: effective options for help-seeking” in Trends and issues in crime and criminal justice, Canberra: Australian Institute of Criminology. vol. 389, 1–6. doi: 10.52922/ti291021

[ref23] Monckton-Smith J. (2021). In control: Dangerous relationships and how they end in murder. London: Bloomsbury Publishing.

[ref24] Morgan K. Buller A. M. Evans M. Trevillion K. Williamson E. Malpass A. (2016). The role of gender, sexuality and context upon help-seeking for intimate partner violence: a synthesis of data across five studies. Aggress. Violent Behav. 31, 136–146. doi: 10.1016/j.avb.2016.09.001

[ref25] Overstreet N. M. Quinn D. M. (2016). “The intimate partner violence stigmatization model and barriers to help seeking” in eds. B. P. John B., A. E. R. Bos Social psychological perspectives on stigma. (Oxford: Routledge), 109–122. 10.1080/01973533.2012.746599 PMC3601798 23524454

[ref26] Patefield W. M. (1981). Algorithm AS 159: an efficient method of generating random R × C tables with given row and column totals. Appl. Stat. 30:91. doi: 10.2307/2346669

[ref27] Peraica T. Kovačić Petrović Z. Barić Ž. Galić R. Kozarić-Kovačić D. (2021). Gender differences among domestic violence help-seekers: socio-demographic characteristics, types and duration of violence, perpetrators, and interventions. J. Fam. Violence 36, 429–442. doi: 10.1007/s10896-020-00207-8

[ref28] Radford L. Hester M. (2006). Mothering through domestic violence. London: Jessica Kingsley Publishers.

[ref29] Robinson S. R. Ravi K. Voth Schrag R. J. (2021). A systematic review of barriers to formal help seeking for adult survivors of IPV in the United States, 2005–2019. Trauma Violence Abuse 22, 1279–1295. doi: 10.1177/1524838020916254, PMID: 32266870

[ref30] Sanz-Barbero B. Briones-Vozmediano E. Otero-García L. Fernández-García C. Vives-Cases C. (2022). Spanish intimate partner violence survivors help-seeking strategies across the life span. J. Interpers. Violence 37, NP8651–NP8669. doi: 10.1177/0886260520976213, PMID: 33289463

[ref31] Schröttle M. Müller U. 2004 Lebenssituation, Sicherheit und Gesundheit von Frauen in Deutschland. Eine repräsentative Untersuchung zu Gewalt gegen Frauen in Deutschland. BMFSFJ. Available online at: https://www.bmfsfj.de/bmfsfj/studie-lebenssituation-sicherheit-und-gesundheit-von-frauen-in-deutschland-80694

[ref32] Stark E. (2007). Coercive control: how men entrap women in personal life. Oxford, UK: Oxford University Press.

[ref33] Taylor J. C. Bates E. A. Colosi A. Creer A. J. (2022). Barriers to men’s help seeking for intimate partner violence. J. Interpers. Violence 37, NP18417–NP18444. doi: 10.1177/08862605211035870, PMID: 34431376 PMC9554285

[ref34] Tjaden P. Thoennes N. (2000). Prevalence and consequences of male-to-female and female-to-male intimate partner violence as measured by the National Violence against Women Survey. Violence Against Women 6, 142–161. doi: 10.1177/10778010022181769

[ref35] Trafford L. (2022). Policing a pandemic: changes in police response to intimate partner violence (IPV) during the first lockdown in England. J. Gend. Based Violence 6, 442–463. doi: 10.1332/239868021X16528069833875

[ref36] Triantafyllou D. Wang C. North C. S. (2019). Correlates of duration of intimate partner violence among women seeking services at a domestic violence support center. J. Interpers. Violence 34, 1127–1138. doi: 10.1177/0886260516647522, PMID: 27150285

[ref37] United Nations. (2025) What is domestic abuse? Available online at: https://www.un.org/en/coronavirus/what-is-domestic-abuse (Accessed October 19, 2025).

[ref9001] WHO. (2024). Factsheets on Violence against Women. Available at: https://www.who.int/news-room/fact-sheets/detail/violence-against-women (Accessed October 14, 2025).

[ref9002] Williams S. L. Kristin D. M. (2008). A Paradox of Support Seeking and Rejection among the Stigmatized. Personal Relationships 15, 493–509. doi: 10.1111/j.1475-6811.2008.00212.x

